# Insights into the Regulation of DMSP Synthesis in the Diatom *Thalassiosira pseudonana* through APR Activity, Proteomics and Gene Expression Analyses on Cells Acclimating to Changes in Salinity, Light and Nitrogen

**DOI:** 10.1371/journal.pone.0094795

**Published:** 2014-04-14

**Authors:** Nicola Louise Kettles, Stanislav Kopriva, Gill Malin

**Affiliations:** 1 Centre for Ocean and Atmospheric Sciences, School of Environmental Sciences, University of East Anglia, Norwich, United Kingdom; 2 John Innes Centre, Norwich, United Kingdom; University of Nottingham, United Kingdom

## Abstract

Despite the importance of dimethylsulphoniopropionate (DMSP) in the global sulphur cycle and climate regulation, the biological pathways underpinning its synthesis in marine phytoplankton remain poorly understood. The intracellular concentration of DMSP increases with increased salinity, increased light intensity and nitrogen starvation in the diatom *Thalassiosira pseudonana*. We used these conditions to investigate DMSP synthesis at the cellular level via analysis of enzyme activity, gene expression and proteome comparison. The activity of the key sulphur assimilatory enzyme, adenosine 5′-phosphosulphate reductase was not coordinated with increasing intracellular DMSP concentration. Under all three treatments coordination in the expression of sulphur assimilation genes was limited to increases in sulphite reductase transcripts. Similarly, proteomic 2D gel analysis only revealed an increase in phosphoenolpyruvate carboxylase following increases in DMSP concentration. Our findings suggest that increased sulphur assimilation might not be required for increased DMSP synthesis, instead the availability of carbon and nitrogen substrates may be important in the regulation of this pathway. This contrasts with the regulation of sulphur metabolism in higher plants, which generally involves up-regulation of several sulphur assimilatory enzymes. In *T. pseudonana* changes relating to sulphur metabolism were specific to the individual treatments and, given that little coordination was seen in transcript and protein responses across the three growth conditions, different patterns of regulation might be responsible for the increase in DMSP concentration seen under each treatment.

## Introduction

Marine phytoplankton play a key role in the global sulphur cycle through the synthesis of dimethylsulphoniopropionate (DMSP), the major precursor of the volatile sulphur compound dimethylsulphide (DMS). DMS transfers sulphur from the oceans, which are a major sulphur reservoir, to the relatively sulphur-limited land [1]. Furthermore, once in the atmosphere, DMS oxidises to form aerosol particles and thereby contributes to cooling the climate, directly through the reflection of solar radiation and indirectly through the formation of cloud condensation nuclei [2]. The global annual flux of DMS from the oceans into the marine atmosphere is estimated at be between 15 and 33 Tg sulphur per year [3] and Gunson et al. [4] used a modelling approach and scenario to demonstrate that halving the DMS flux could have a significant effect on radiative forcing, increasing surface temperatures by around 1.6°C. More recent debate highlights additional phytoplankton-derived compounds that may also produce particles and reflect radiation from the Sun back into space [5].

Considerable variability in DMSP production has been observed between phytoplankton taxa [6]. The Dinophyceae and the Prymnesiophyceae are the highest DMSP producers with intracellular concentrations in excess of several hundred mmol l−1 in some species, although diatoms and members of other groups can also produce significant amounts. The chlorophytes on the other hand produce very little DMSP [6], with the exception of seaweeds such as *Ulva lactuca* [7]. In addition to taxonomy, external factors, such as nutrient availability, salinity and temperature can cause variations in intracellular DMSP concentration. Multiple cellular roles have been proposed for DMSP, including as an osmolyte [7], [8], cryoprotectant [9], grazing deterrent [10], antioxidant defence [11] and as an ‘overflow metabolite’ in dissipation of excess energy, carbon and reducing equivalents when growth and photosynthesis are unbalanced [12]. The primary role of DMSP in the cell, however, remains uncertain and might vary within the lifespan of a cell and between species.

**Figure 1:**
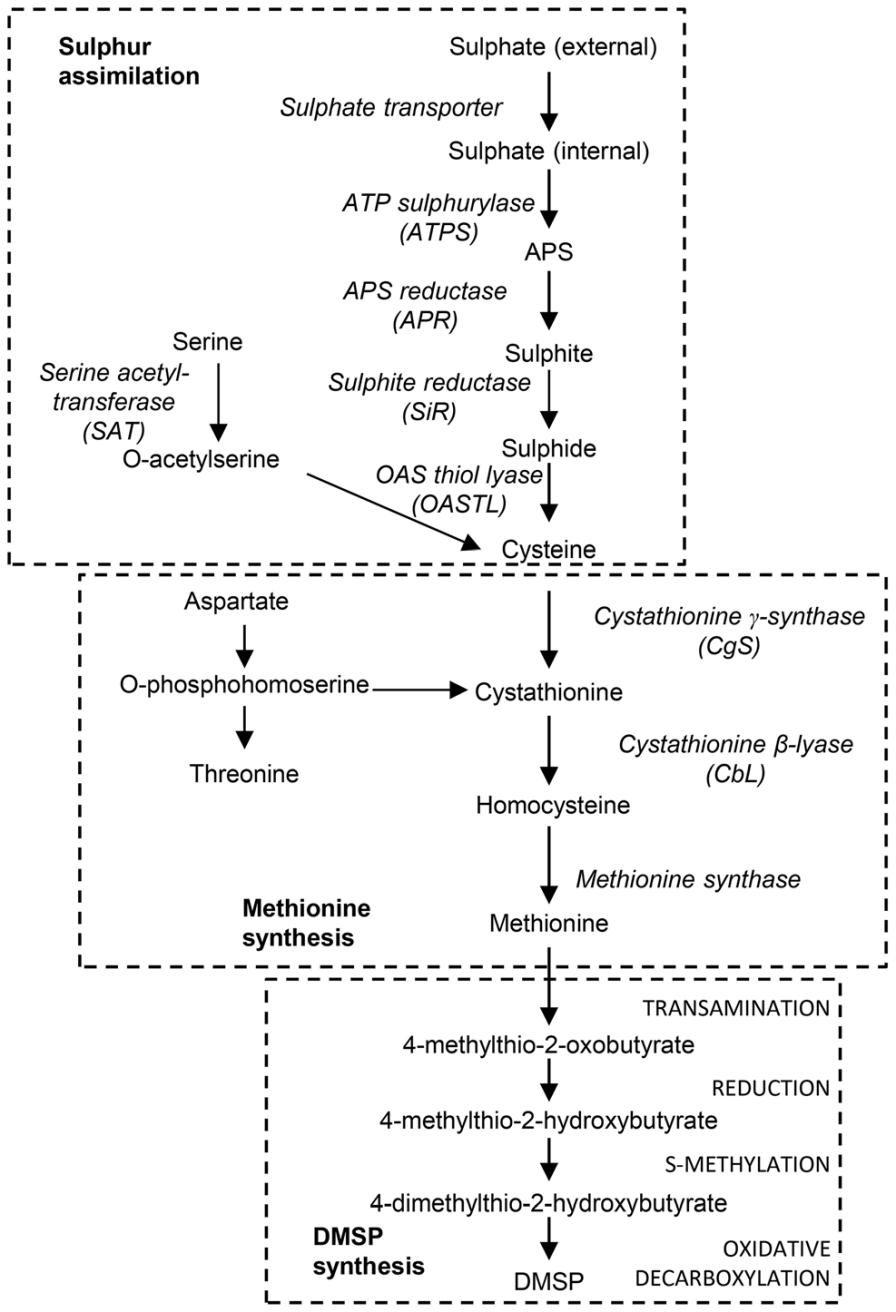
A proposed pathway of sulphate assimilation and DMSP biosynthesis in algae. Enzymes between the methionine and DMSP have not yet been identified. The putative reactions proposed by [13] are shown in capitals.

DMSP is generated from methionine [13] and Gage et al. [14] used ^35^S-labelling to determine the steps converting this amino acid to DMSP in the green macroalga *Ulva intestinalis*. Methionine first undergoes transamination to 4-methylthio-2-oxobutyrate (MTOB) and then reduction to 4-methylthio-2-hydroxybutyrate (MTHB), before *S*-methylation to 4-dimethylsulphonio-2-hydroxybutyrate (DMSHB) and finally oxidative decarboxylation to DMSP (Figure 1) [14]. The key intermediate, DMSHB, has also been identified in the marine microalgae *Emiliania huxleyi*, *Tetraselmis sp.* and the diatom *Melosira nummuloides*, indicating that the same pathway exists in diverse algal classes [14]. The activities of putative, substrate-specific enzymes for the first three steps of this pathway were measured in *U. intestinalis* [15], although the enzymes have not been identified.

Despite the importance of DMSP production in the sulphur cycle and climate regulation, and the availability of genome sequences for several DMSP producing phytoplankton, there has been limited research into basic sulphur metabolism in this group. We have very little direct knowledge of this pathway in most phytoplankton species and know almost nothing about its regulation. A detailed understanding of the biological mechanism of DMSP production is required to improve our ability to predict how environmental factors might affect this process. Comparing cell protein and transcript abundance has the potential to offer new insight into the pathway of sulphur assimilation and DMSP biosynthesis, and its regulation, in marine phytoplankton.

Diatoms are not generally considered major DMSP producers, but the synthesis of DMSP by members of this group has been shown to be up-regulated under certain growth conditions [6], [16]. For example, Bucciarelli and Sunda [16] measured increased DMSP concentration in the diatom *Thalassiosira pseudonana* under nitrate, phosphate and silicate limitation. Given that diatoms are one of the most abundant groups of phytoplankton, accounting for approximately 20% global primary production, they might therefore make a greater contribution to DMSP production than suggested by early studies on actively growing, nutrient-replete batch cultures [6]. In addition, the ability to up-regulate DMSP production is a useful characteristic for investigating the regulation of its synthesis pathway. *T. pseudonana* is also an excellent model for diatom biology because its genome has been sequenced [17] and there are various molecular tools for this species. Our aim was to use *T. pseudonana* to address the control of DMSP synthesis at a cellular level.

We analysed the proteome response of *T. pseudonana* to increased salinity and increased light intensity, two conditions that we have confirmed to induce DMSP synthesis in this species, using 2-dimensional gel electrophoresis. We then compared these findings to our previous study on the proteome response of *T. pseudonana* to nitrogen starvation [18], a growth condition that also increases intracellular DMSP concentration in this species [16]. The overall hypothesis was that enzymes related to DMSP synthesis would be found amongst proteins that increased or decreased in abundance across all three of these growth conditions. In addition, since DMSP is an important sink for reduced sulphur, we also compared the transcript abundance of genes involved in the uptake and assimilation of sulphate and measured the activity of the key enzyme of the pathway, adenosine 5′-phosphosulphate reductase (APR) under increased salinity, increased light intensity and nitrogen starvation.

## Materials and Methods

### Culturing

Axenic cultures of *T. pseudonana* (CCMP 1335, National Centre for Marine Algae and Microbiota, Bigelow, USA) were grown in batch culture in ESAW (enriched seawater, artificial water) medium [19] at 15oC with a 14∶10 light:dark cycle. Unless otherwise stated, cultures were grown at 115 µmol photons m−2 s−1 based on an immersed measurement with a Scalar PAR Irradiance Sensor QSL 2101 (Biospherical Instruments Inc., San Diego, USA). Cultures were regularly checked for bacterial contamination by 4′,6-diamidino-2-phenylindoledihydrochloride (DAPI) staining [20]. Cell number and volume were measured with a Beckman Coulter Multisizer 3 Analyser (Beckman Coulter Ltd, High Wycombe, UK) and variable to maximum fluorescence ratio (F_v_/F_m_) with a Walz Phyto-Pam phytoplankton analyser (Heinz Walz GmbH, Effeltrich, Germany). Experiments were conducted in triplicate for control and treated cultures. All measurements and samples for further analysis were collected 3 hours into the light period.

### Alteration of Salinity

The salinity of ESAW growth medium was adapted by either increasing or decreasing the concentrations of all salts in the ESAW artificial seawater base recipe [19] with the exception of bicarbonate, because changes in its concentration affected culture growth rate and yield. *T. pseudonana* cells, acclimated to a salinity of 10 psu for a minimum of four rounds of subculture (approximately 4 weeks) prior to the experiment, were grown in triplicate to ca. 1×106 cells ml−1. At this point the cultures were divided equally and an equal volume of medium with a salinity of either 10 psu to maintain the salinity, or 60 psu, to achieve a final salinity of 35, was added to each portion. Samples were taken for transcript and proteome comparison 48 h after the salinity change, on day 4.

The salinity was adjusted by the dilution of all salts because it is a more environmentally relevant treatment than only changing levels of sodium chloride, however, this also decreased the concentration of sulphate from 25 mM, as in standard ESAW medium, to 5 mM. This decrease in sulphate concentration without changing overall salinity was tested and found to have no effect on growth rate or intracellular DMSP concentration (Figure S1).

### Alteration of Light Intensity

*T. pseudonana* cultures acclimated to a reduced light intensity of 50 µmol photons m−2 s−1 (achieved using a neutral density filter) for a minimum of four rounds of subculturing (approximately 4 weeks) were grown to a density of ca. 5×105 cells ml−1. Cultures were then either kept at 50 µmol photons m−2 s−1 or exposed to a high light intensity of 1000 µmol photons m−2 s−1. Samples were taken for transcript and proteome comparison 48 h after cultures were exposed to increased light intensity, on day 8.

### Nitrogen Starvation

As described in Hockin et al. [18] *T. pseudonana* cultures were started with initial concentrations of 550 µM (standard ESAW) or 30 µM nitrate. Cultures with an initial concentration of 30 µM or 550 µM nitrate became yield-limited at ca. 1×106 cell ml−1and ca. 2×106 cell ml−1, respectively. Samples were taken for transcript and proteome comparison on day 3 of the experiment.

### Intracellular DMSP Concentration

Intracellular DMSP concentration was measured using headspace gas chromatography, developed by [21]. Depending on the cell density, 3 to 7 ml of culture was collected by filtration onto 25 mm nucleopore track-etch membranes with a 1 µm pore (Whatman) at a vacuum pressure no greater than 15 kPa. Samples were then handled as described in [22], with the exception that 150 µl of headspace gas was sampled and injected into the GC. Technical triplicates were conducted for this method.

### Gene Expression Analysis

*T. pseudonana* cells were collected by quick filtration of 250 ml culture aliquots onto 47 mm diameter 1 µm nucleopore membranes (Whatman) at a vacuum pressure of 35 kPa. Filters were immediately frozen in liquid nitrogen and stored at −80°C. Cells were washed from the membrane with 500 µl buffer (80 mM Tris pH 9, 5% SDS, 150 mM LiCl, 50 mM EDTA), and homogenised. Total RNA was extracted by phenol/chlorophorm and LiCl precipitation.

Quantitative real-time RT-PCR (qPCR) was performed using gene-specific primers with efficiencies between 87 and 105% (Table S1) and the fluorescent intercalating dye SYBR Green (Sigma-Aldrich), in a DNA engine OPTICON2 continuous fluorescence detector (Bio-Rad). The programme steps were: 2 min at 95°C, then 40 cycles consisting of 15 s at 95°C, 15 s at 60°C, 30 s at 72°C and 10 min at 72°C, followed by a standard dissociation protocol to ensure that each amplicon was a single product. The relative difference in transcript abundance between the control and treatment samples was calculated using the ΔΔCt method [23]. Beta-tubulin (TUB3) was used as the reference gene, and its stability was confirmed using geNorm software [24]. The RT-PCR reactions were performed in triplicate for each of the three independent biological replicates.

### APR Activity

Depending on the cell density, 5 to 15 ml of culture was centrifuged and resuspended in 500 µl of extraction buffer (45.45 mM Na/KPO_4_, 27 mM Na_2_SO_3_, 0.45 mM AMP, and 9.1 mM DTE). The cells were disrupted by sonication (three times for 10 sec at ca. 15 microns on ice with a Soniprep 150 probe; MSE, London, UK) and APR activity was measured as the production of [35 S]sulphite assayed as acid volatile radioactivity formed in the presence of [35 S]APS and dithioerythritol (DTE) as reductant [25]. APR activity was normalized to protein concentration, which was measured according to the Bradford assay [26] using a Bio-Rad Protein kit (Figure S2).

### Proteome comparison by 2-dimensional gel electrophoresis

The cultures were collected by filtration of multiple 250 ml aliquots onto, 47 mm diameter nucleopore membranes with a 1 µm pore size (Whatman) at a vacuum pressure of 35 kPa. Filters were immediately frozen in liquid nitrogen and stored at −80°C. For each of the three independent cultures, proteins from two filters were extracted, and 100 ìg protein, as quantified with an Ettan 2-D Quant kit (GE Healthcare, Chalfont, UK) was subjected to 2-dimensional (2-D) gel electrophoresis and imaged exactly as described in [18]. Gel images were compared using Progenesis SameSpots analysis software (v4.1; Nonlinear Dynamics Ltd, Newcastle Upon Tyne, UK), which includes automatic background subtraction and normalization. Protein spots with altered levels of expression (1.5 fold change and q<0.05, t-test corrected for false discovery rate (FDR) using the Benjamini Hochberg procedure [27]) under treated versus control conditions, in all three growth conditions, were excised from the gel using a ProPick excision robot (Genomic Solutions). The excised proteins were then manually in-gel trypsin digested and analysed by peptide mass fingerprinting [28], as described in [18].

## Results and Discussion

### Growth Conditions Affecting DMSP

**Figure 2:**
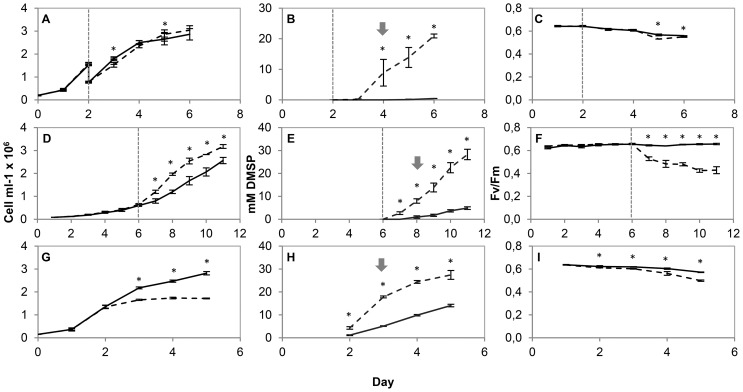
Regulation of DMSP concentration. The effect of increased salinity (A,B,C; solid line indicates cultures maintained at 10 psu and dashed line indicates cultures transferred to 35 psu), increased light intensity (D,E,F,; solid line indicates cultures maintained at 50 µmols m^−2^ s^−1^ and dashed line indicates cultures transferred to 1000 µmols m^−2^ s^−1^) and nitrogen starvation (G,H,I; solid line indicates nitrogen replete cultures and dashed line indicates nitrogen starved cultures) on cell number (A,D,G), the DMSP concentration (B,E,H) and Fv/Fm (C,F,I) of *Thalassiosira pseudonana*. Results are shown as means ± standard deviation from 3 independent cultures. The vertical line indicates the point at which the salinity/light intensity of the cultures was adjusted. Asterisks show t-test, P<0.05. Arrows indicate the point at which cultures were sampled for proteomics and gene expression analysis.

The identification of conditions that increase the concentration of DMSP in *T. pseudonana* cells was a critical first step in enabling us to study cells with up-regulated DMSP synthesis. We confirmed that the intracellular DMSP concentration of *T. pseudonana* increases with increased salinity, increased light intensity and nitrogen starvation. Cells transferred from 10 psu to 35 psu had an intracellular DMSP concentration of 8.9 mM after 48 hours whilst its concentration in cells maintained at 10 psu remained below the level of detection (Figure 2A,B). When samples were collected for proteome and transcriptional analysis, 48 h after the increase in salinity, no difference in Fv/Fm between the two treatment groups was observed (Figure 2C). Exposure to a high light intensity of 1000 µmol photons m−2 s−1 for 48 hours lead to a 8-fold higher intracellular DMSP concentration compared to cells maintained at 50 µmol photons m−2 s−1 (Figure 2D,E). The Fv/Fm of cultures exposed to a higher light intensity decreased within 24 h and remained below that of cultures maintained at a lower light intensity at 48 h when samples were collected for proteome and transcriptional analysis (Figure 2F). The intracellular DMSP concentration of nitrogen starved *T. pseudonana* on day 3 was 3.5-fold higher than that of nitrogen replete cultures (initial nitrate concentrations 30 µM and 550 µM respectively; Figure 2G,H). On day 3, when samples were collected for proteome and transcriptional analysis, there was a small, but significant (t-test, P<0.05) decrease in the Fv/Fm of nitrogen starved cultures compared to nitrogen replete cultures (Figure 2I). In addition to providing a useful tool for our study, these increases in cellular DMSP, in a single microalgal species under three diverse growth treatments (osmotic stress, oxidative stress and nutrient depletion) highlight the multifunctional role of DMSP in the cell.

### APR Activity

DMSP is a product of sulphur metabolism and the assimilation of this element may therefore limit DMSP synthesis. The reduction of 5′-adenylylsulphate (APS), catalysed by the enzyme APR, is the key point in regulation of the pathway of sulphate assimilation in higher plants [29] and is regulated in a demand driven manner by the thiols glutathione and cysteine [30], [31], amino compounds [32], [33], carbohydrates [34], [35] and hormones [36], [37]. To test whether APR might also have a regulatory role in the biosynthesis of DMSP in *T. pseudonana*, its activity was measured under increased salinity, increased light intensity and nitrogen starvation.

**Figure 3:**
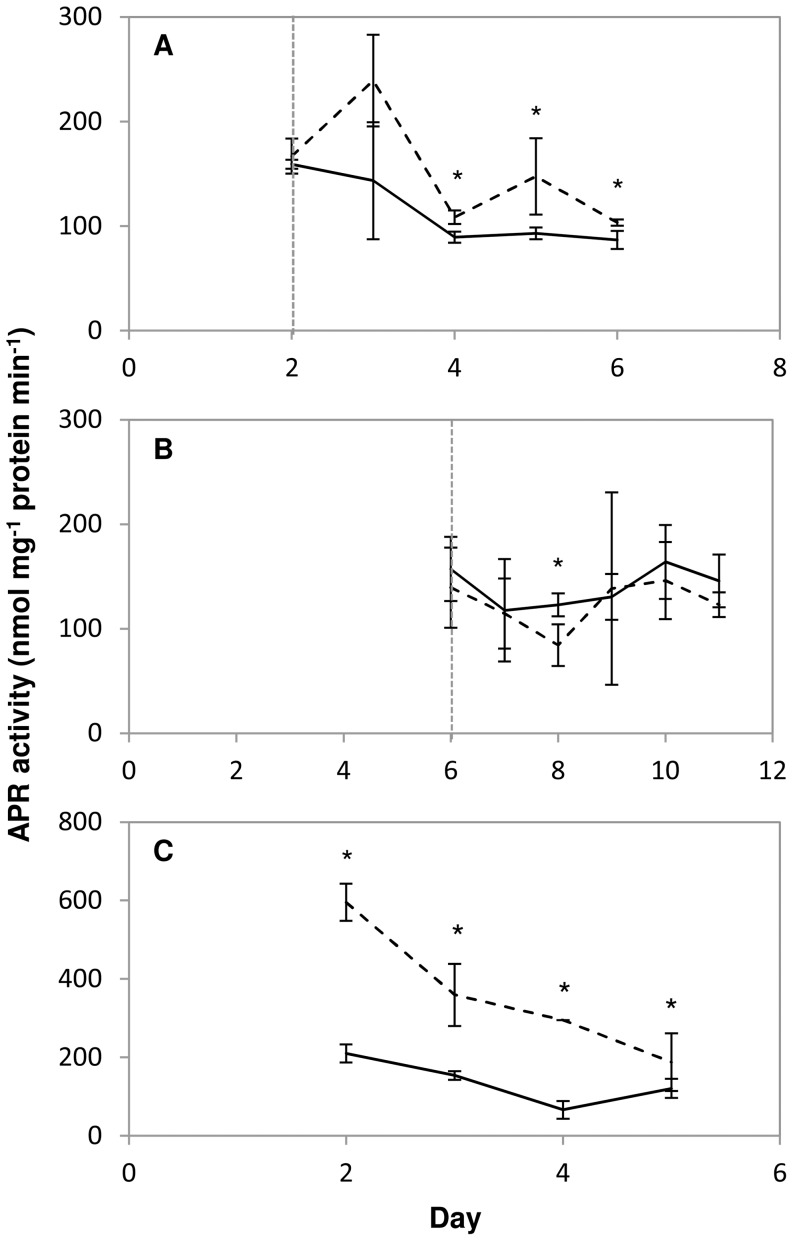
Regulation of APR activity. The effect of increased salinity (A; solid line, cultures maintained at 10 psu; dashed line, cultures transferred to 35 psu), increased light intensity (B; solid line, cultures maintained at 50 µmol m^−2^ s^−1^; dashed line, cultures transferred to 1000 µmol m^−2^ s^−1^) and nitrogen starvation (C; solid line, nitrogen replete cultures; dashed line, nitrogen starved cultures) on APR activity in *Thalassiosira pseudonana*. Results are shown as means ± standard deviation from 3 independent cultures. The vertical line indicates the point at which the salinity/light intensity of the cultures was adjusted. Asterisks mark significantly different values (P<0.05, T-test).

The APR activity measured in *T. pseudonana* cultures adjusted from a salinity of 10 psu to 35 psu increased slightly, but significantly after 48 h (Figure 3A). For the remainder of the experiment APR activity was only a little higher than in the cultures kept at 10 psu, despite the substantial increase in intracellular DMSP concentration of cultures adjusted to a salinity of 35, increasing from below the level of detection to 20 mM between day 3 and day 6 (Figure 2B). There was no significant difference in cellular protein content between the two salinity treatments (Figure S2) There was no difference in APR activity in *T. pseudonana* cultures exposed to a high light intensity of 1000 µmol m−2 s−1 compared to those kept at 50 µmol m−2 s−1 (Figure 3B), whilst again there was a clear increase in intracellular DMSP concentration (Figure 2E). Again, no significant difference in cellular protein content was observed (Figure S2). Under nitrogen starvation APR activity was 2.8-fold higher than in nitrogen replete cultures on day 2 (Figure 3C), the first time point measured, when an increase in intracellular DMSP had already been detected (Figure 2H). However, whilst the DMSP concentration of the nitrogen starved cells continued to increase throughout the experiment the APR activity gradually decreased to the level of the nitrogen replete cultures. As was found in our previous study [18] cellular protein content was decreased under nitrogen starvation, compared to nitrogen replete cells. The APR activities measured in the different cultures under normal nitrogen supply were between 66 and 209 nmol min−1 mg−1 protein depending on growth stage and condition of the cultures (Figure 3C). This is comparable to the APR activity reported for the diatoms *Thalassiosira weissflogii* and *Thalassiosira oceanica* which ranged from 80 to 200 nmol min−1 mg−1 protein [38] and, interestingly, two orders of magnitude higher than the activities typically found in plants.

Gao et al. [38] also found that APR activity decreased through the growth curve despite a progressive increase in DMSP content per cell under nitrogen limitation in the chlorophyte microalga *T. subcordiformis*. In addition, Bochenek et al. [22] showed similar decrease in APR activity during the course of batch cultures of the haptophyte *Emiliania huxleyi*, another high DMSP producer. These results suggest that there is no clear relationship between intracellular DMSP concentration and APR activity in *T. pseudonana*. The initial higher APR activity under nitrogen starvation might be required for a short term increase in sulphur assimilation to initially increase DMSP synthesis, but subsequently the sulphur for DMSP synthesis might be made available by other cell processes. In accordance, the lack of regulation of APR activity by increased light intensity suggests that APR is not critical for increased DMSP biosynthesis. Given the very high APR activity levels it is possible that this is adequate to provide reduced sulphur for DMSP synthesis without a need for further up-regulation. Gao et al. [38] reported that APR activity of the dinoflagellate *Heterocapsa triquetra*, was only 0.5 to 5 nmol min−1 mg−1, which is considerably lower than that found for diatoms in that study and confirmed here. Thus, as *H. triquetra* has a high intracellular DMSP concentration of approximately 300 mM [39], compared to a maximum of 15 to 20 mM for *T. pseudonana,* regulation of APR activity may be uncoupled from control of DMSP synthesis. Alternatively, there might be a fundamental divergence in the regulation of sulphur metabolism between these two groups of phytoplankton. This is plausible, as there is evidently a substantial difference in regulation of APR in *T. pseudonana* and plants. While in the diatom APR was not significantly affected by increased salinity and was higher in nitrogen starved cells than in controls, in Arabidopsis the enzyme is induced by salt and repressed by nitrogen starvation [33], [40].

### Expression of Genes involved in Sulphur Assimilation

**Figure 4:**
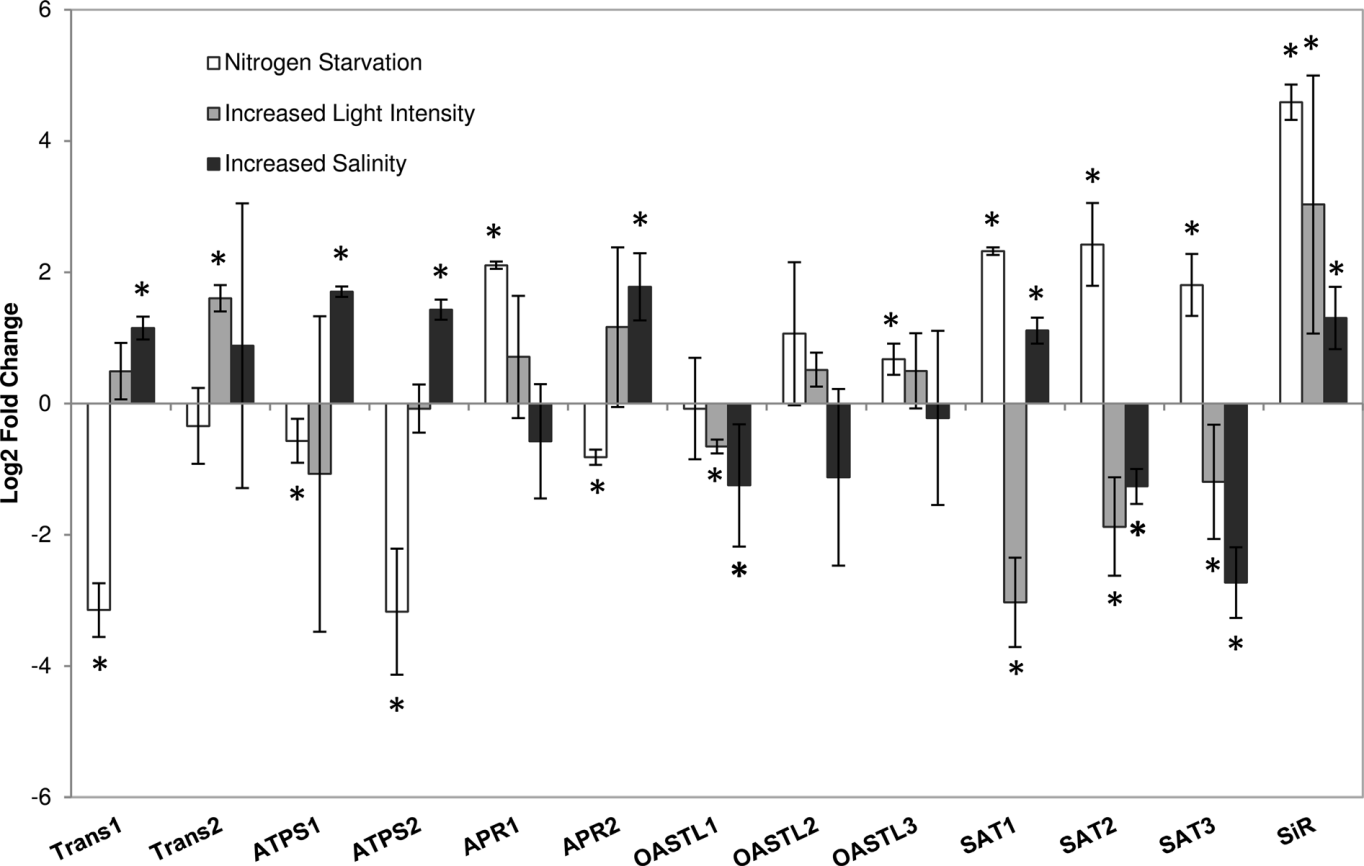
Regulation of gene expression. The relative fold change in the transcript levels of genes involved in sulphur assimilation in *Thalassiosira pseudonana* exposed to nitrogen starvation, increased salinity and increased light intensity quantified by qRT-PCR. Gene abbreviations are as follows: Sulphate transporter (Trans), ATP sulphurylase (ATPS), APS reductase (APR), OAS thiollyase (OASTL), Serine acetyltransferase (SAT), Sulphite reductase (SiR). Numbers denote different isoforms of the enzymes (for gene IDs see Table S1). Results are shown as means ± standard deviation from 3 independent cultures. Asterisks mark values significantly different between treatments and control at P<0.05 (T-test).

Since APR does not seem to be important for control of DMSP synthesis, the contribution of other components of sulphur metabolism was assessed by comparing transcript levels of genes associated with the assimilation of sulphate to cysteine under increased salinity, increased light intensity and nitrogen starvation. Surprisingly, little coordination was found in the transcript responses to the three treatments, including the two isoforms of APR (Figure 4). In contrast, in plants these genes are often upregulated coordinately e.g. following jasmonate and salt treatment, or with the exception of ATP sulphurylase by sulphur starvation [29]. Transcript levels of *APR1* (gene ID 35690) increased under nitrogen starvation, but not with increased salinity or light intensity, whereas *APR2* (ID 24887) transcript levels did increase significantly with increased salinity, but not with the other two treatments. This suggests that different APR isoforms may be responsible for the initial increase in APR activity seen with nitrogen starvation and with the increase with increased salinity. The lack of change in APR transcripts under increased light intensity corresponds with the clear lack of regulation of APR activity under this treatment.

Interestingly, the transcript levels of all three serine acetyltransferase (SAT, gene IDs 38294, 37497, 16842) genes increased 5-, 5.7-, and 3.6-fold, respectively, in *T. pseudonana* under nitrogen starvation (Figure 4). In plants SAT is often associated with the regulation of sulphur assimilation through the production of O-acetylserine (OAS), as modulation of SAT expression affects the concentration of cysteine and glutathione [41], [42]. Thus OAS synthesis might be limiting for cysteine synthesis under nitrogen starvation, and consequently DMSP production in *T. pseudonana*. However, only *SAT1* (ID 38294) expression increased with increased salinity and no changes in any SAT isoforms were seen with increased light intensity. Therefore, a universal role for SAT in control of sulphate assimilation in this diatom species is unlikely.

The only gene up-regulated under all three growth conditions was sulphite reductase (SiR; gene ID 31984; Figure 4), which could therefore represent an important point of regulation in diatom sulphur assimilation. APR activity in diatoms is about two orders of magnitude higher than in plants and could therefore be too high to effectively control the flux through sulphate assimilation. Thus the control might move to another component of the pathway possibly by limiting cysteine synthesis. SiR is a good candidate for such control point as reduction of its expression in Arabidopsis limits growth [43]. Alternatively, in addition to the different responses of APR activity, the general lack of coordination in this pathway, across the treatments indicates that sulphur assimilation might not limit DMSP production. This is supported by our finding that reducing sulphate availability in ESAW medium from 25 mM to 5 mM had no effect on DMSP concentration in *T. pseudonana* (Figure S1) and, contrary to what has been seen for other marine phytoplankton species, no negative effect on growth [44]. With *Emiliania huxleyi* Bochenek et al. [22] found that reducing sulphate concentration to 5 mM reduced growth rate by 50% and intracellular DMSP concentration by 60%. It should be noted that *E. huxleyi* has an intracellular DMSP concentration several fold higher than that of *T. pseudonana* and so is likely to have higher sulphur requirements than the diatom [45]. To add complexity to the regulation of sulphur assimilation and DMSP synthesis, at a number of steps in the pathway there are multiple isoforms that can catalyse the reaction, and these might well have different kinetic properties and/or be localised to different cellular compartments. The differential regulation of isoforms seen here thus warrants further investigation.

### Identification of Proteins associated with DMSP synthesis

**Figure 5:**
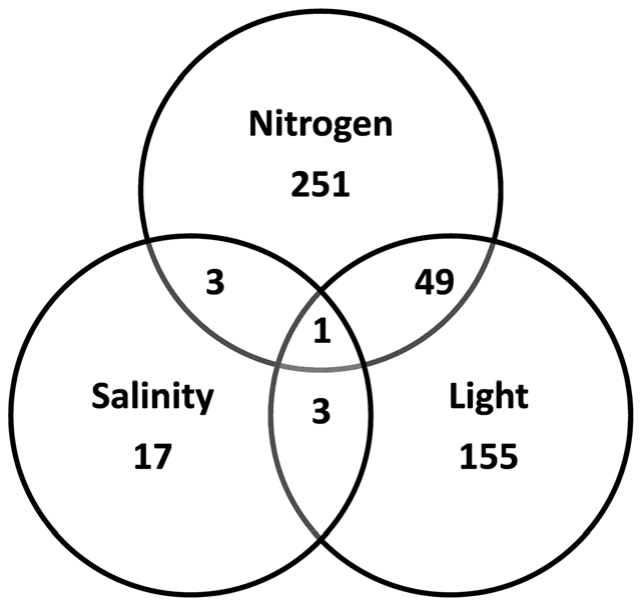
Global proteome changes. Venn diagram showing the number of protein spots identified by 2-dimensional gel electrophoresis, changing in relative abundance by more than 1.5-fold (t-test, FDR, q<0.05) in *Thalassiosira pseudonana* under nitrogen starvation, increased salinity and increased light intensity.

In an unbiased approach to identify enzymes involved in DMSP biosynthesis, we identified changes in the proteomes of *T. pseudonana* cells in response to increased salinity and light intensity and compared these to our previous dataset on the response of *T. pseudonana* to nitrogen starvation [18]. The separation of protein extracts by 2-dimensional gel electrophoresis yielded 3310 distinguishable protein spots. There were 479 spots that changed by more than 1.5-fold in relative abundance with q<0.05 (determined by t-test and corrected by FDR) under any of the three treatments. We had hypothesised that any protein found to change in abundance in the same direction under all three treatments would potentially be involved in DMSP biosynthesis, however we found that most of the changes were treatment specific, with very few proteins changing in abundance under multiple treatments. Only 1 spot changed under all three treatments, however, the 1,5-fold cut-off was met only in the nitrogen and high light dataset. (Figure 5). This spot (ID: 00569) belongs to a horizontal chain of spots that all increased under all three growth conditions that increased intracellular DMSP concentration. Although the fold change was not greater than 1.5-fold in all cases, at least one spot in the chain increased by 1.5-fold under each treatment. These spots were interesting because they could be same protein possibly separated due to posttranslational modifications that affect the proteins isoelectric point. Five spots were picked and indeed all identified as a phosphoenolpyruvate carboxylase (PEPC; ProtID 268546; table 1).

**Table 1:** Proteins related to sulphur assimilation, methionine metabolism, and photosynthesis changing in abundance under nitrogen starvation (N), increased salinity (S) or increased light intensity (L).

Protein ID	Protein Name	Fold Change			FDR (q)		
		N	S	L	N	S	L
260934	Branched-chain-amino-acid aminotransferase	6.4	1.3	−1.2	0.0062	0.2804	0.2638
270365	Sulphite reductase^mbc^	1.6	1.2	1.1	0.0187	0.2804	0.2998
20797	S-adenosylmethionine (SAM)- dependent Methyltransferase^mbc^	−1.7	3.6	1.2	0.0516	0.0559	0.1854
		−3.0	3.6	1.3	0.0492	0.0574	0.1156
644	Adenosine kinase	1.1	2.5	1.2	0.2455	0.0179	0.0558
21815	Adenosylmethionine synthetase	−1.4	2.9	1.1	0.0469	0.0277	0.25564
27273	Methylenetetrahydrofolate reductase	1.0	2.0	1.5	0.4515	0.0582	0.0257
28496	Adenosylhomocysteinase	−1.7	2.6	1.3	0.021	0.0569	0.0765
25402	Fucoxanthin chl a/c light-harvesting protein	−1.0	1.2	−3.6	0.424	0.3975	0.0118
22747	Fucoxanthin chlorophyll a/c light-harvesting protein	−1.0	1.0	−3.5	0.4793	0.4825	0.0127
bd1048	Photosystem I iron-sulphur centre^MB^	1.0	−1.2	−2.6	0.3337	0.0957	0.0159
30385	Fucoxanthin chlorophyll a/c protein-LI818 clad	−1.2	−1.2	−2.5	0.2049	0.3020	0.0127
24080	Fucoxanthin chl a/c light-harvesting protein	−1.1	1.2	−2.4	0.346	0.405	0.0285
38583	Fucoxanthin chlorophyll a/c protein	1.0	−1.4	−2.4	0.4518	0.0408	0.0118
268546	Phosphoenolpyruvate carboxylase	2.5	1.7	1.4	0.0161	0.1789	0.0547
		2.0	1.5	1.4	0.0499	0.2037	0.0473
		1.8	1.3	1.4	0.0208	0.1746	0.0244
		1.6^z^	1.7^z^	1.5^z^	0.023	0.0661	0.0801
		2.5	1.2	1.6	0.0062	0.0408	0.0181

m Manual Annotation.

b Supported by BlastP (E<3×10^−32^).

c Supported by conserved domains identified through Pfam.

z Combined with another protein making fold change imprecise.

Protein names are based on UniProtKB unless otherwise stated and Protein IDs are from the Joint Genome Institute *T. pseudonana* genome version 3 (http://genome.jgi-psf.org/Thaps3/Thaps3.home.html).

Since the response of the *T. pseudonana* proteome was quite different between the different treatments we picked the spots with the strongest changes from each treatment. For the salinity and light treatments the 10 spots with the greatest fold change, and sufficient quantity were picked from the gel, and in the nitrogen starvation study all spots that changed were picked [18]. In total 135 spots were picked, from which 16 could not be identified, resulting in identification of 84 unique proteins, by MALDI-TOF MS analysis of the tryptic digests, that changed in relative abundance under one or more treatment (Table S2), including proteins related to sulphur metabolism. It is important to note that the absence of a protein among the analysed spots does not prove its stable abundance as not all regulated proteins can be identified by 2-dimensional electrophoresis. For example gels are not optimised for membrane bound proteins and very low abundance proteins might also not be detected.

#### Increased Salinity

**Figure 6:**
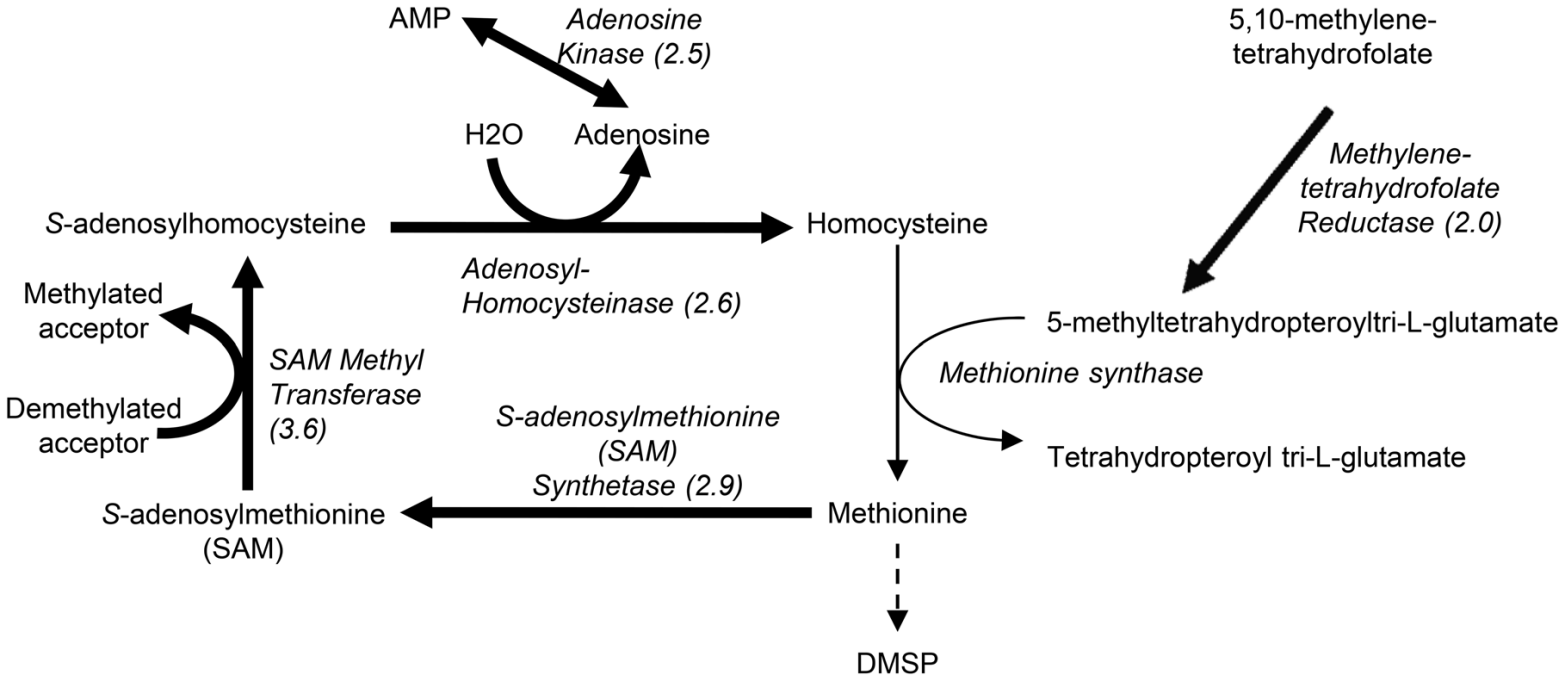
A scheme of the active methyl cycle. Enzymes that increased in abundance with increased salinity in *T. pseudonana* are marked with bold arrows.

Among proteins which increased in abundance specifically with increased salinity (Tables 1, S2), the proteins involved in the active methyl cycle (Figure 6), which salvages methionine from methyl transferase reactions, were most prominent. An S-adenosylmethionine (SAM) synthetase (ProtID 21815), which catalyses the formation of SAM from methionine, increased by 2.9-fold. SAM is used as a methyl donor in a wide variety of methyltransferase reactions and accordingly a 3.6-fold increase in the abundance of a SAM-dependent methyltransferase (ProtID 20797) was measured. Enzymes of this family have a diverse range of functions [46] including synthesis of various metabolites. The SAM-dependent methyltransferase identified (ProtID 20797) has similarities to a sarcosine/dimethylglycine methyltransferase (BlastP E = 3×10−32) which provides an alternative route for synthesis of the osmolyte glycine betaine (GBT) through the methylation of glycine. This metabolic route of GBT synthesis is distinct from the pathway of choline oxidation found in higher plants and it has been identified in a number of halotolerant bacteria and cyanobacteria [47], [48] and the red alga *Galdieria sulphuraria* [49].

The S-adenosylhomocysteine produced by such methyltransferase activity can be recycled to homocysteine through the action of adenosylhomocysteinase; this enzyme (ProtID 28496) increased 2.6-fold with increased salinity. Adenosine is the second product of this reaction and, correspondingly, an adenosine kinase (ProtID 644), which catalyses the reversible phosphorylation of adenosine to adenosine monophosphate, increased 2.5-fold. This enzyme might remove adenosine, thereby promoting the activity of homocysteinase. Together the enzymes of the active methyl cycle have the potential to influence the free methionine pool and thereby its availability for DMSP synthesis under increased salinity. An increase in the abundance of enzymes associated with the active methyl cycle with increased salinity has also been reported in the sea-ice diatom *Fragilariopsis cylindrus* [50].

The abundance of methylenetetrahydrofolate reductase (ProtID 27273) increased 2-fold under increased salinity. This enzyme reduces 5,10-methylenetetrafolate to 5-methyltetrahydropteroyl tri-L-glutamate, which donates its methyl group for synthesis of methionine from homocysteine. The disruption of this enzyme in the bacterium *Streptomyces lividans* leads to methionine auxotrophy [51]. Methylenetetrahydrofolate reductase might therefore be limiting methionine synthesis and DMSP production in *T. pseudonana*.

#### Increased Light Intensity

Methylenetetrahydrofolate reductase (ProtID 27273) increased 1.5-fold also under increased light intensity, suggesting that there may be some similarity in the response of sulphur metabolism to increased salinity and increased light intensity. However, enzymes of the active methyl cycle remained unchanged.

The most obvious response of *T. pseudonana* to increased light intensity is the degradation of photosynthetic proteins; a well-defined response in photosynthetic organisms [52]–[55]. Five light harvesting proteins (ProtIDs 25402, 22747, 30385, 24080, 38583) and one photosystem I centre (ProtID bd1563) decreased in abundance relative to the control (Table 1), suggesting that this is also the case in the response of *T. pseudonana* to increased light intensity, although reduced translation or transcription cannot be ruled out. The degradation of photosynthetic proteins could increase the availability of amino acids such as cysteine and methionine for DMSP synthesis. Gröne and Kirst [56] first demonstrated that protease inhibitor delayed DMSP accumulation in the Prasinophyte *Tetraselmis (Platymonas) subcordiformis* suggesting that protein degradation has a role in the regulation of DMSP synthesis. Futhermore, Lyonn et al [50] have also proposed that degradation of light harvesting proteins could be a potential source of cysteine and methionine for DMSP induction based on their study of the *F. cylindrus* proteome response to salinity.

#### Nitrogen Starvation

Under nitrogen starvation a decrease in cellular protein level was measured in *T. pseudonana* and there was also evidence of increased protein catabolism [18], which would influence the amino acid pools in the same way as the degradation of photosynthetic proteins under increased light intensity. On the other hand, the proteome response to nitrogen starvation was quite different to that of increased salinity. The abundance of methylenetetrahydrofolate reductase was unchanged suggesting that this enzyme does not contribute to the increase in DMSP measured under this treatment. In addition the abundance of many enzymes of the active methyl cycle were reduced under nitrogen starvation, with the SAM-dependent methyltransferase identified in this study decreased by 2.1-fold. This is not entirely surprising since a reciprocal relationship that is dependent on nitrogen availability has been found between cellular DMSP and the nitrogen based osmolyte, glycine betaine (GBT) in continuous cultures of *T. pseudonana* [57]. However, this again suggests that, whilst the active methyl cycle might have a role in increasing the free methionine pool and DMSP synthesis with increased salinity, this is not the case regarding nitrogen starvation.

The protein with the greatest increase in abundance identified in this study was a branched chain aminotransferase (ProtID 260934) that increased 6.4-fold under nitrogen starvation, however it did not change under increased salinity or light intensity. This enzyme might be a candidate for the first step of DMSP biosynthesis, catalysing the production of MTOB from methionine by transamination. However, further research would be required to confirm this, such as substrate specificity testing and demonstrating that over expression or silencing of the gene has an effect on DMSP synthesis. If this enzyme was found to catalyse the transamination of Methionine to MTOB it would raise questions as to why it did not increase with increased salinity or light intensity.

The only enzyme found to increase in abundance by more than 1.5-fold under all three treatments that increased the intracellular DMSP concentration of *T. pseudonana* was a PEPC (ProtID 268546). Oxaloacetate, produced by PEPC, is used in the synthesis of aspartate, which is a precursor of methionine. O-phosphohomoserine (OPH; Figure 1) is an intermediate between aspartate and methionine, and is the substrate of both cystathionine ã-synthase (CgS), forming cystathionine, and threonine synthase, forming threonine. The balance of these processes is an important point of regulation in methionine synthesis in higher plants [58]. It is plausible that increased PEPC activity is a response to increased demand for aspartate and DMSP synthesis. In plants, particularly those with C4 photosynthesis, PEPC is also induced by salt stress, it is linked to pH homeostasis and carbon/nitrogen balance [59]. Similar roles in diatoms cannot be excluded, indeed, the increased expression of PEPC and aminotransferases can be used for intracellular nitrogen recycling to maintain C/N balance [60]. As *T. pseudonana* expresses all enzymes of the C_4_ photosynthesis cycle [61] it can be speculated that the increase in PEPC might serve as a CO_2_ pump to reduce photorespiration and loss of C and N. Should these be the mechanisms of action of PEPC, the increase DMSP might be just a mechanism of dissipation of excess sulphur. However, this would most probably trigger a repression of sulphate uptake and assimilation, which was not the case.

### What does this tell us about the regulation of DMSP production?

The lack of coordination in transcript level throughout the pathway of sulphur assimilation in these experiments suggests that sulphur does not limit DMSP production in *T. pseudonana*, as is the case for sulphur compounds in higher plants. This would not be entirely surprising since marine algae evolved in a very different sulphur environment to terrestrial plants [44]. The ocean is a sulphur reservoir where this nutrient is unlikely to limit productivity, whereas in terrestrial habitats sulphur availability does limit growth. The APR activities measured here in *T. pseudonana*, and in other algal species [38], are more than two orders of magnitude higher than in plants. Gao et al. [38] suggest that these algal species have a high capacity to assimilate sulphate and may build up intracellular stores that are used in DMSP production rather than newly assimilated sulphate.

Another possible reason for the lack of coordination in the proteome response of *T.pseudonana* under conditions that increase in DMSP synthesis is that this process might not depend on the synthesis of new proteins. There are many possible levels of regulation, so that possibly allosteric regulation(s) may control DMSP synthesis, as is the case for methionine synthesis. Threonine synthase, which competes with CgS for OPH, is under allosteric control and this regulates the flow of carbon into threonine or methionine.

There are some patterns in the transcript and protein responses to the treatments used here that could highlight potential points of regulation other than sulphate assimilation. Interestingly, a number of these changes occurred in the branches that bring carbon and nitrogen skeletons to the central pathway of sulphur assimilation. These include increased transcript levels of SAT under nitrogen starvation, increased methyltetrahydrofolate reductase protein abundance under increased salinity and light intensity, and increase PEPC protein abundance under all three growth conditions. This may suggest that carbon and/or nitrogen skeletons limit DMSP synthesis rather than sulphur. Only transcript levels of SiR were increased under all three growth conditions suggesting that this enzyme might be a key controlling point of sulphur assimilation in diatoms rather than APR as in higher plants.

In a study that focussed on the effect of increased salinity on DMSP synthesis in the sea-ice diatom *Fragilariopsis cylindrus*, Lyon et al. [50] identified candidate enzymes for the four classes of enzymes that Gage et al. [14] proposed to catalyse the conversion of methionine to DMSP (aminotransferase, reductase, methyl transferase, decarboxylase). Although their activity has yet to be confirmed, it is interesting that using a comparable proteomic approach, but looking for proteins that changed in three different treatments that increase intracellular DMSP concentration, these proteins were not identified. This could be due to differential regulation between these species, indeed these diatoms inhabit quite different environments. It is also possible that these proteins did increase under increased salinity, but were not among the highest changing and therefore not picked, since not all protein spots were analysed in this study. However, neither were they identified in our nitrogen starvation treatment where all the proteins that changed significantly were analysed. It is possible that the changes in protein abundance observed by [50] are specific to increased DMSP content with increased salinity and that alternative processes might be responsible for the increase in DMSP under nitrogen starvation. There was a very high consistency between the 2D gels analysed in our study, however, we cannot dismiss the possibility that an increased number of replicates might lead to identification of additional significant differences.

It is thought that DMSP has multiple functions within the cell, and indeed, in the experiments here it might be considered to be used as an osmolyte, antioxidant and possibly as an overflow metabolite. Perhaps the lack of coordination in the response of the pathway of sulphur assimilation to these growth conditions indicates that different regulatory mechanisms, each potentially leading to an increase in the methionine pool, might relate to its different roles in the cell under the different conditions. Under increased salinity the production of glycine betaine via the methylation of glycine, might affect the methionine pool through the active methyl cycle; of which many of the enzymes were seen to increase in abundance. The increase in the abundance of methylenetetrahydrofolate reductase under increased salinity and light intensity is likely to increase 5-methyltetrahydropteroyl tri-L-glutamate supply for methionine biosynthesis, thereby increasing the availability of this amino acid. Methionine availability could be increased under increased light intensity and nitrogen starvation by protein degradation. Perhaps there is no individual limiting step or ‘on-switch’ for DMSP synthesis, instead under different conditions different components could be limiting. This might depend on the carbon and nitrogen status of the cell, each of which will be different under the three conditions tested here.

## Supporting Information

Figure S1 **Effect of sulphate limitation on** ***Thalassiosira pseudonana*** **.**
(PDF)

Figure S2 **Cellular protein content of** ***T. pseudonana*** **grown under different salinities, light intensity and nitrogen availability.**
(PDF)

Figure S3 **Examples of 2D gels.**
(PDF)

Table S1 **Primer sequences used for quantitative real-time PCR.**
(PDF)

Table S2 **Proteins changed in abundance under increased salinity, increased light intensity or nitrogen starvation.**
(XLSX)

## References

[1] Lovelock JE, Maggs RJ, Rasmussen RA (1972) Atmospheric dimethyl sulfide and natural sulfur cycle. Nature 237: 452–453.

[2] Charlson RJ, Lovelock JE, Andreae MO, Warren SG (1987) Oceanic phytoplankton, atmopspheric sulphur, cloud albedo and climate. Nature 326: 655–661.

[3] Kettle AJ, Andreae MO (2000) Flux of dimethylsulfide from the oceans: A comparison of updated data sets and flux models. J Geophys Res-Atmosph 105: 26793–26808.

[4] Gunson JR, Spall SA, Anderson TR, Jones A, Totterdell IJ, et al (2006) Climate sensitivity to ocean dimethylsulphide emissions. Geophys Res Lett 33: L07701.

[5] Ayers GP, Cainey JM (2007) The CLAW hypothesis: a review of the major developments. Environ Chem 4: 366–374.

[6] Keller M, Bellows W, Guillard R (1989) Dimethyl sulfide production in marine phytoplankton. In: Saltzman E, Cooper W, eds. Biogenic Sulfur in the Environment. Washington, DC: American Chemical Society, 167–182.

[7] Dickson DMJ, Wyn Jones RG, Davenport J (1980) Steady state osmotic adaption in *Ulva lactuca* . Planta 150: 158–165. 24306591 10.1007/BF00582360

[8] Vairavamurthy A, Andreae MO, Iverson RL (1985) Biosynthesis of dimethylsulfide and dimethylpropiothetin by *Hymenomonas carterae* in relation to sulfur source and salinity variations. Limnol Ocean 30: 59–70.

[9] Karsten U, Kuck K, Vogt C, Kirst G (1996) Dimethylsulfoniopropionate production in phototrophic organisms and its physiological function as a cryoprotectant. In: Kiene R, Visscher P, Keller M, Kirst G, eds. Biological and Environmental Chemistry of DMSP and Related Sulfonium Compounds.New York: Plenum, 143–153.

[10] Wolfe GV, Steinke M, Kirst GO (1997) Grazing-activated chemical defence in unicellular marine algae. Nature 387: 894–897.

[11] Sunda W, Kieber DJ, Kiene RP, Huntsman S (2002) An antioxidant function for DMSP and DMS in marine algae. Nature 418: 317–320. 12124622 10.1038/nature00851

[12] Stefels J (2000) Physiological aspects of the production and conversion of DMSP in marine algae and higher plants. J Sea Res 43: 183–197.

[13] Greene RC (1962) Biosynthesis of dimethyl-β-propiothetin. J Biol Chem 237: 2251–2254. 13901535

[14] Gage DA, Rhodes D, Nolte KD, Hicks WA, Leustek T, et al (1997) A new route for synthesis of dimethylsulphoniopropionate in marine algae. Nature 387: 891–894. 9202120 10.1038/43160

[15] Summers PS, Nolte KD, Cooper AJL, Borgeas H, Leustek T, et al (1998) Identification and Stereospecificity of the First Three Enzymes of 3-Dimethylsulfoniopropionate Biosynthesis in a Chlorophyte Alga. Plant Physiol 116: 369–378.

[16] Bucciarelli E, Sunda WG (2003) Influence of CO2, nitrate, phosphate, and silicate limitation on intracellular dimethylsulfoniopropionate in batch cultures of the coastal diatom Thalassiosira pseudonana. Limnol Ocean 48: 2256–2265.

[17] Armbrust EV, Berges JA, Bowler C, Green BR, Martinez D, et al (2004) The genome of the diatom *Thalassiosira pseudonana*: ecology, evolution, and metabolism. Science 306: 79–86. 15459382 10.1126/science.1101156

[18] Hockin NL, Mock T, Mulholland F, Kopriva S, Malin G (2012) The response of diatom central carbon metabolism to nitrogen starvation is different from that of green algae and higher plants. Plant Physiol 158: 299–312. 22065419 10.1104/pp.111.184333 PMC3252072

[19] Harrison J, Berges A (2005) Marine culture medium. In: Andersen RA, ed. Algal Culturing Techniques. San Diego: Academic Press, 21–33.

[20] Porter KG, Feig YS (1980) The use of DAPI for identifying and counting aquatic microflora. Limnol Ocean 25: 943–948.

[21] Steinke M, Malin G, Turner SM, Liss PS (2000) Determinations of dimethylsulphoniopropionate (DMSP) lyase activity using headspace analysis of dimethylsulphide (DMS). J Sea Res 43: 233–244.

[22] Bochenek M, Etherington GJ, Koprivova A, Mugford ST, Bell TG, et al (2013) Transcriptome analysis of the sulfate deficiency response in the marine microalga *Emiliania huxleyi* . New Phytol 199: 650–662. 23692606 10.1111/nph.12303

[23] Pfaffl MW (2001) A new mathematical model for relative quantification in real-time RT-PCR. Nucleic Acids Res 29: e45. 11328886 10.1093/nar/29.9.e45 PMC55695

[24] Vandesompele J, De Preter K, Pattyn F, Poppe B, Van Roy N, et al (2002) Accurate normalization of real-time quantitative RT-PCR data by geometric averaging of multiple internal control genes. Gen Biol 3: RESEARCH0034. 10.1186/gb-2002-3-7-research0034 PMC126239 12184808

[25] Kopriva S, Fritzemeier K, Wiedemann G, Reski R (2007) The putative moss 3′-phosphoadenosine-5′-phosphosulfate reductase is a novel form of adenosine-5′-phosphosulfate reductase without an iron-sulfur cluster. J Biol Chem 282: 22930–22938. 17519237 10.1074/jbc.M702522200

[26] Bradford MM (1976) A Rapid and Sensitive Method for the Quantitation of Microgram Quantities of Protein Utilizing the Principle of Protein-Dye Binding. Anal Biochem 72: 248–254. 942051 10.1016/0003-2697(76)90527-3

[27] Benjamini Y, Hochberg Y (1995) Controlling the false discovery rate: a practical and powerful approach to multiple testing. J Roy Stat Soc B 57: 289–300.

[28] Pappin DJC, Hojrup P, Bleasby AJ (1993) Rapid identification of proteins by peptide-mass fingerprinting. Anal Biochem 3: 327–332. 10.1016/0960-9822(93)90195-t 15335725

[29] Takahashi H, Kopriva S, Giordano M, Saito K, Hell R (2011) Sulfur Assimilation in Photosynthetic Organisms: Molecular Functions and Regulations of Transporters and Assimilatory Enzymes. Annu Rev Plant Biol 62: 157–184. 21370978 10.1146/annurev-arplant-042110-103921

[30] Bick JA, Setterdahl AT, Knaff DB, Chen Y, Pitcher LH, et al (2001) Regulation of the Plant-type 5′-Adenylyl Sulfate Reductase by Oxidative Stress. Biochemistry 40: 9040–9048. 11467967 10.1021/bi010518v

[31] Vauclare P, Kopriva S, Fell D, Suter M, Sticher L, et al (2002) Flux control of sulphate assimilation in *Arabidopsis thaliana*: adenosine 5′-phosphosulphate reductase is more susceptible than ATP sulphurylase to negative control by thiols. Plant J 31: 729–740. 12220264 10.1046/j.1365-313x.2002.01391.x

[32] Neuenschwander U, Suter M, Brunold C (1991) Regulation of Sulfate Assimilation by Light and O-Acetyl-l-Serine in *Lemna minor* L. Plant Physiol 97: 253–258. 16668378 10.1104/pp.97.1.253 PMC1080991

[33] Koprivova A, Suter M, Den Camp RO, Brunold C, Kopriva S (2000) Regulation of sulfate assimilation by nitrogen in Arabidopsis. Plant Physiol 122: 737–746. 10712537 10.1104/pp.122.3.737 PMC58909

[34] Kopriva S, Muheim R, Koprivova A, Trachsel N, Catalano C, et al (1999) Light regulation of assimilatory sulphate reduction in *Arabidopsis thaliana* . Plant J 20: 37–44. 10571863 10.1046/j.1365-313x.1999.00573.x

[35] Kopriva S, Suter M, Von Ballmoos P, Hesse H, Krahenbuhl U, et al (2002) Interaction of sulfate assimilation with carbon and nitrogen metabolism in *Lemna minor* . Plant Physiol 130: 1406–1413. 12428005 10.1104/pp.007773 PMC166659

[36] Harada E, Kusano T, Sano H (2000) Differential expression of genes encoding enzymes involved in sulfur assimilation pathways in response to wounding and jasmonate in *Arabidopsis thaliana* . J Plant Physiol 156: 272–276.

[37] Ohkama N, Takei K, Sakakibara H, Hayashi H, Yoneyama T, et al (2002) Regulation of sulfur-responsive gene expression by exogenously applied cytokinins in *Arabidopsis thaliana* . Plant Cell Physiol 43: 1493–1501. 12514246 10.1093/pcp/pcf183

[38] Gao Y, Schofield OM, Leustek T (2000) Characterization of sulfate assimilation in marine algae focusing on the enzyme 5′-adenylylsulfate reductase. Plant Physiol 123: 1087–1096. 10889258 10.1104/pp.123.3.1087 PMC59072

[39] Caruana AMN, Steinke M, Turner SM, Malin G (2012) Concentrations of dimethylsulphoniopropionate and activities of dimethylsulphide-producing enzymes in batch cultures of nine dinoflagellate species. Biogeochemistry 110: 87–107.

[40] Koprivova A, North KA, Kopriva S (2008) Complex signaling network in regulation of adenosine 5′-phosphosulfate reductase by salt stress in Arabidopsis roots. Plant Physiol 146: 1408–1420. 18218969 10.1104/pp.107.113175 PMC2259037

[41] Harms K, Von Ballmoos P, Brunold C, Höfgen R, Hesse H (2000) Expression of a bacterial serine acetyltransferase in transgenic potato plants leads to increased levels of cysteine and glutathione. Plant J 22: 335–343. 10849350 10.1046/j.1365-313x.2000.00743.x

[42] Haas FH, Heeg C, Queiroz R, Bauer A, Wirtz M, et al (2008) Mitochondrial serine acetyltransferase functions as a pacemaker of cysteine synthesis in plant cells. Plant Physiol 148: 1055–1067. 18753283 10.1104/pp.108.125237 PMC2556817

[43] Khan MS, Haas FH, Samami AA, Gholami AM, Bauer A, et al (2010) Sulfite Reductase Defines a Newly Discovered Bottleneck for Assimilatory Sulfate Reduction and is Essential For Growth and Development in *Arabidopsis thaliana* . Plant Cell 22: 1216–1231. 20424176 10.1105/tpc.110.074088 PMC2879758

[44] Ratti S, Knoll AH, Giordano M (2011) Did sulfate availability facilitate the evolutionary expansion of chlorophyll a+c phytoplankton in the oceans? Geobiology 9: 301–312. 21627761 10.1111/j.1472-4669.2011.00284.x

[45] Franklin DJ, Airs RL, Fernandes M, Bell TG, Bongaerts RJ, et al (2012) Identification of senescence and death in *Emiliania huxleyi* and *Thalassiosira pseudonana*: Cell staining, chlorophyll alterations, and dimethylsulfoniopropionate (DMSP) metabolism. Limnol Ocean 57: 305–317.

[46] Loenen W (2006) S-adenosylmethionine: jack of all trades and master of everything? Biochem Soc Trans 34: 330–333. 16545107 10.1042/BST20060330

[47] Nyyssölä A, Kerovuo J, Kaukinen P, Von Weymarn N, Reinikainen T (2000) Extreme Halophiles Synthesize Betaine from Glycine by Methylation. J Biol Chem 275: 22196–22201. 10896953 10.1074/jbc.M910111199

[48] Waditee R, Tanaka Y, Aoki K, Hibino T, Jikuya H, et al (2003) Isolation and Functional Characterization of N-Methyltransferases That Catalyze Betaine Synthesis from Glycine in a Halotolerant Photosynthetic Organism *Aphanothece halophytica* . J Biol Chem 278: 4932–4942. 12466265 10.1074/jbc.M210970200

[49] McCoy JG, Bailey LJ, Ng YH, Bingman CA, Wrobel R, et al (2009) Discovery of sarcosine dimethylglycine methyltransferase from *Galdieria sulphuraria* . Proteins-Struct Funct Bioinf 74: 368–377. 10.1002/prot.22147 PMC2742680 18623062

[50] Lyon BR, Lee PA, Bennett JM, DiTullio GR, Janech MG (2011) Proteomic analysis of a sea-ice diatom: salinity acclimation provides new insight into the dimethylsulfoniopropionate production pathway. Plant Physiol 157: 1926–1941. 22034629 10.1104/pp.111.185025 PMC3327215

[51] Blanco J, Coque JJR, Martin JF (1998) The Folate Branch of the Methionine Biosynthesis Pathway in *Streptomyces lividans*: Disruption of the 5,10-Methylenetetrahydrofolate Reductase Gene Leads to Methionine Auxotrophy. J Bacteriol 180: 1586–1591. 9515933 10.1128/jb.180.6.1586-1591.1998 PMC107064

[52] Hui Y, Jie W, Carpentier R (2000) Degradation of the Photosystem I Complex During Photoinhibition. Photochem Photobiol 72: 508–512. 11045722 10.1562/0031-8655(2000)072<0508:dotpic>2.0.co;2

[53] Yamamoto Y (2001) Quality Control of Photosystem II. Plant Cell Physiol 42: 121–128. 11230565 10.1093/pcp/pce022

[54] Zolla L, Rinalducci S (2002) Involvement of Active Oxygen Species in Degradation of Light-Harvesting Proteins under Light Stresses. Biochemistry 41: 14391–14402. 12450406 10.1021/bi0265776

[55] Rajagopal S, Joly D, Gauthier A, Beauregard M, Carpentier R (2005) Protective effect of active oxygen scavengers on protein degradation and photochemical function in photosystem I submembrane fractions during light stress. FEBS J 272: 892–902. 15691324 10.1111/j.1742-4658.2004.04512.x

[56] Gröne T, Kirst GO (1992) The effect of nitrogen deficiency, methionine and inhibitors of methionine metabolism on the DMSP contents of *Tetraselmis subcordiformis* (Stein). Mar Biol 112: 497–503.

[57] Keller MD, Kiene RP, Matrai PA, Bellows WK (1999) Production of glycine betaine and dimethylsulfoniopropionate in marine phytoplankton. II. N-limited chemostat cultures. Marine Biol 135: 249–257.

[58] Hesse H, Kreft O, Maimann S, Zeh M, Hoefgen R (2004) Current understanding of the regulation of methionine biosynthesis in plants. J Exp Bot 55: 1799–1808. 15234989 10.1093/jxb/erh139

[59] Doubnerová V, Ryšlavá H (2011) What can enzymes of C4 photosynthesis do for C3 plants under stress? Plant Sci 180: 575–583. 21421406 10.1016/j.plantsci.2010.12.005

[60] Nunn BL, Faux JF, Hippmann AA, Maldonado MT, Harvey HR, et al (2013) Diatom proteomics reveals unique acclimation strategies to mitigate Fe limitation. PLoS One 8: e75653. 24146769 10.1371/journal.pone.0075653 PMC3797725

[61] Nunn BL, Aker JR, Shaffer SA, Tsai S, Strzepek RF, et al (2009) Deciphering diatom biochemical pathways via whole-cell proteomics. Aquat Microb Ecol 55: 241–253. 19829762 10.3354/ame01284 PMC2761042

