# Supplementary material for: Insights into the Regulation of DMSP Synthesis in the Diatom Thalassiosira pseudonana through APR Activity, Proteomics and Gene Expression Analyses on Cells Acclimating to Changes in Salinity, Light and Nitrogen
Source: PLoS One. 2014 Apr 14;9(4):e94795. doi: 10.1371/journal.pone.0094795 (PMC3986220; doi:10.1371/journal.pone.0094795)
Supplement: Figure S1 — Effect of sulphate limitation on Thalassiosira pseudonana . (PDF) [file pone.0094795.s001.pdf]

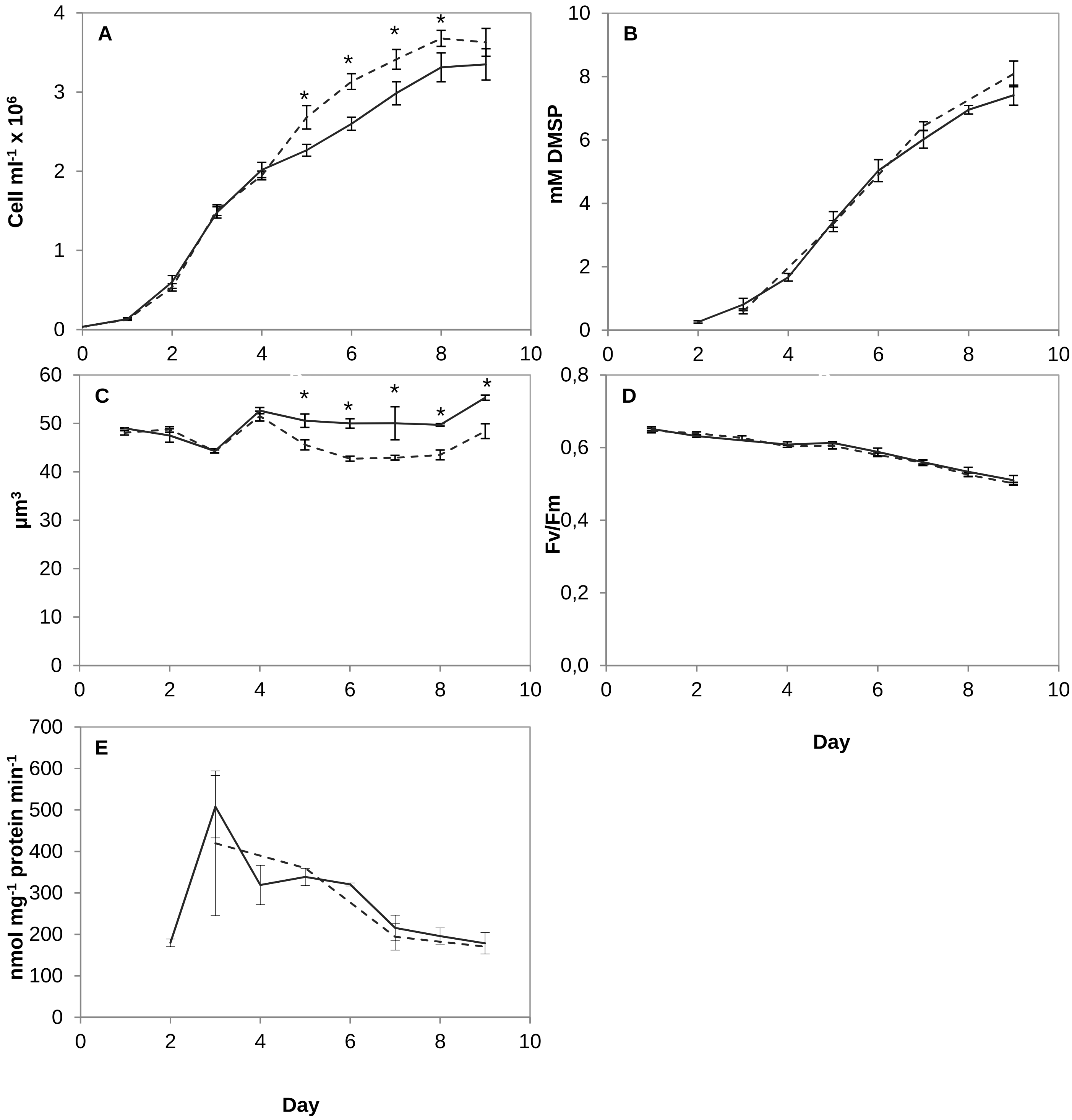

Supplementary Figure S1. Effect of sulphate limitation on *Thalassiosira pseudonana*. **A**. cell number **B**. intracellular DMSP concentration, **C**. volume per cell, **D**. Fv/Fm (The ratio of variable to maximum fluorescence), **E**. APR activity of *Thalassiosira pseudonana* cultures with an initial sulphate concentration of either 5 mM (dashed line) or 25 mM (solid line). Results are shown as means ± standard deviation from 3 independent cultures. Asterisks mark values significantly different ($P<0.05$, T-test) between the 5 mM and 25 mM cultures.
