# Supplementary material for: Insights into the Regulation of DMSP Synthesis in the Diatom Thalassiosira pseudonana through APR Activity, Proteomics and Gene Expression Analyses on Cells Acclimating to Changes in Salinity, Light and Nitrogen
Source: PLoS One. 2014 Apr 14;9(4):e94795. doi: 10.1371/journal.pone.0094795 (PMC3986220; doi:10.1371/journal.pone.0094795)
Supplement: Figure S2 — Cellular protein content of T. pseudonana grown under different salinities, light intensity and nitrogen availability. (PDF) [file pone.0094795.s002.pdf]

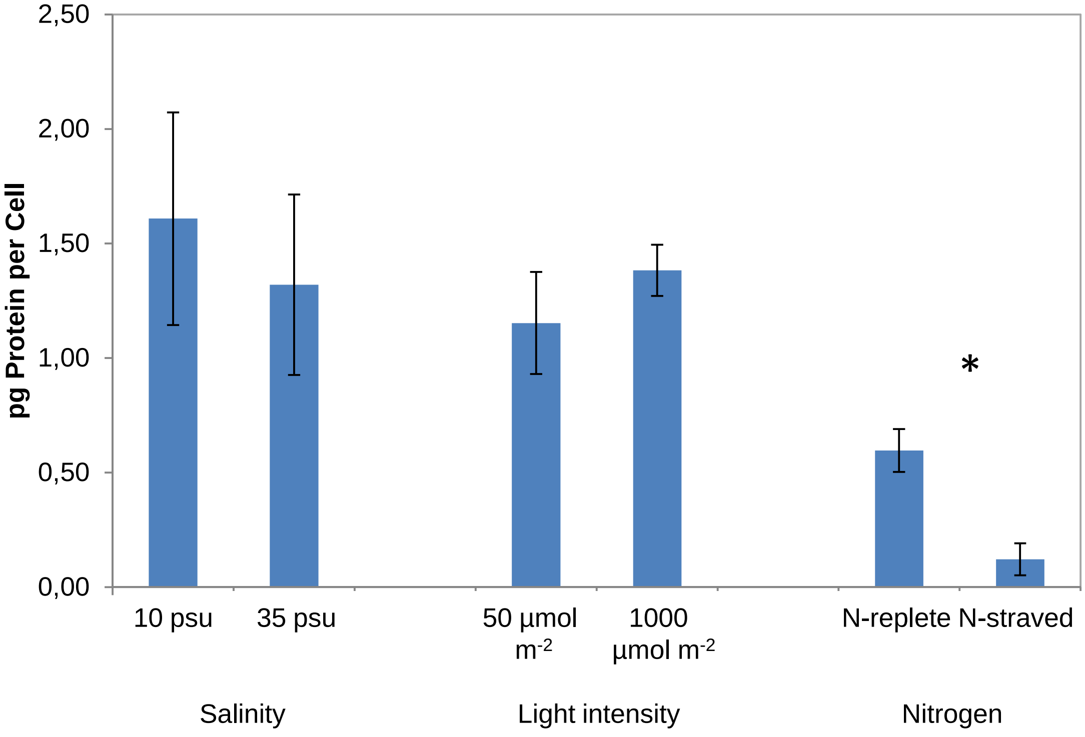

Supplementary Figure S2. Cellular protein content of *T. pseudonana* grown under different salinities, light intensity and nitrogen availability at the point when cultures were sampled for further proteome and transcript analysis (salinity day 4, light day 8 and nitrogen day 3). Asterisks mark values significantly different ($P<0.05$, T-test) between the nitrogen replete and N-deplete cultures.
