# Supplementary material for: Insights into the Regulation of DMSP Synthesis in the Diatom Thalassiosira pseudonana through APR Activity, Proteomics and Gene Expression Analyses on Cells Acclimating to Changes in Salinity, Light and Nitrogen
Source: PLoS One. 2014 Apr 14;9(4):e94795. doi: 10.1371/journal.pone.0094795 (PMC3986220; doi:10.1371/journal.pone.0094795)
Supplement: Figure S3 — Examples of 2D gels. (PDF) [file pone.0094795.s003.pdf]

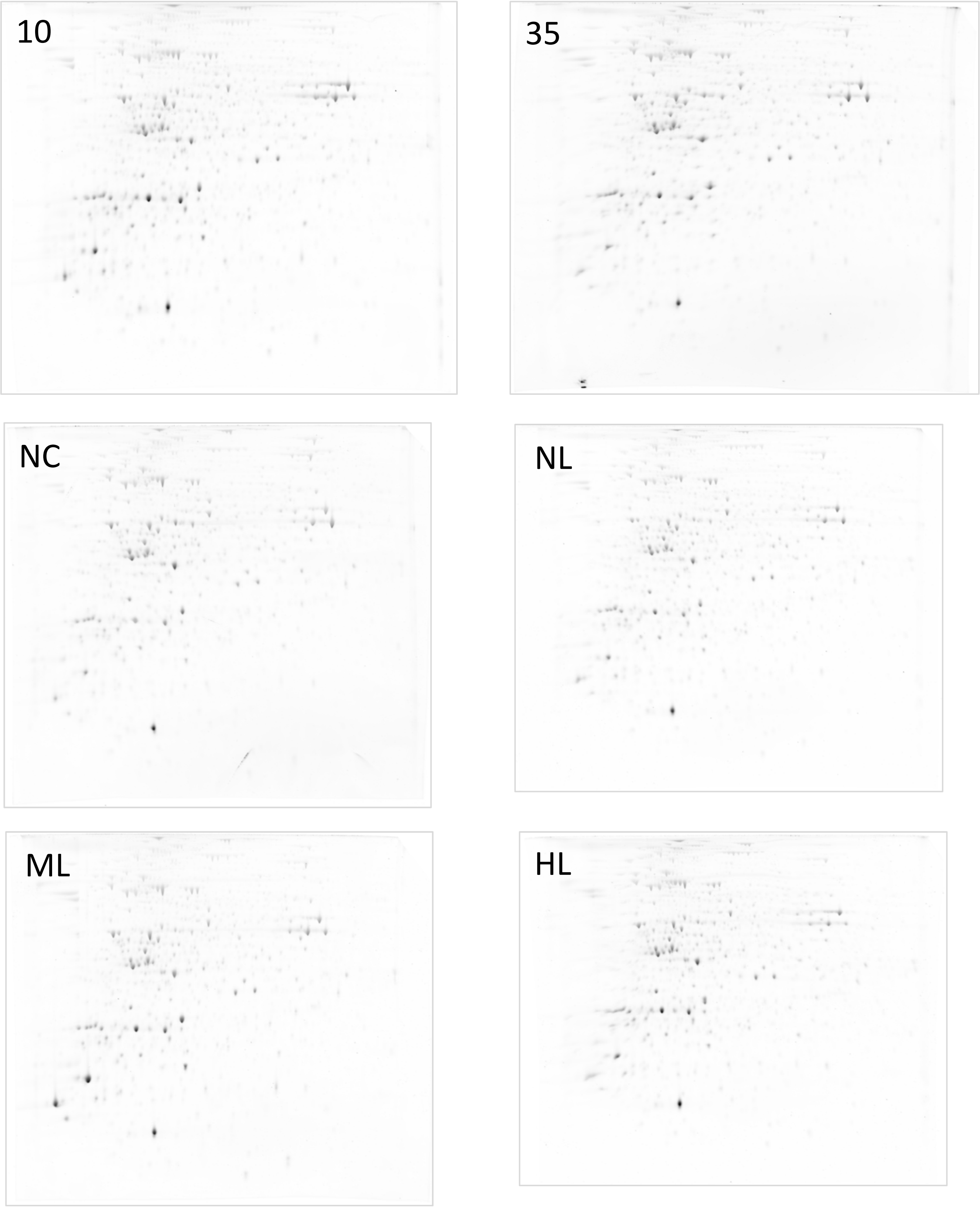

Supplementary Figure S3. Examples of 2 dimensional gels used in the comparison of the proteome of *T. pseudonana* grown under low and normal salinity (10, 35), nitrogen replete and nitrogen limiting (NC, NL) and moderate and high light (ML, HL) conditions.
