# Supplementary material for: Insights into the Regulation of DMSP Synthesis in the Diatom Thalassiosira pseudonana through APR Activity, Proteomics and Gene Expression Analyses on Cells Acclimating to Changes in Salinity, Light and Nitrogen
Source: PLoS One. 2014 Apr 14;9(4):e94795. doi: 10.1371/journal.pone.0094795 (PMC3986220; doi:10.1371/journal.pone.0094795)
Supplement: Table S1 — Primer sequences used for quantitative real-time PCR. (PDF) [file pone.0094795.s004.pdf]

Supplementary Table S1. Primers used in qRT-PCR studies. Length (L), melting temperature (MT), % GC content (GC), product size (PS), efficiency (E).

	Gene	Gene ID	Sequence	L	MT	GC	PS	E
Beta Tubulin	TUB3	31569	L:AAGGAGGTGGATGATGAGATG	21	58.95	47.62	152	2.03
			R:ATGCAGGTGGAGTTACCAATG	21	59.87	47.62		
Sulphate Transporter 1	Trans1	261147	L:CATTGTCGGTATCGCATCTG	20	60.10	50.00	137	2.02
			R:TCGACATTGAACCACTCACTG	21	59.74	47.62		
Sulphate Transporter 2	Trans2	263781	L:TGTTCACCATCTTGTTGACGAC	22	61.00	45.45	121	1.97
			R:GCACTGATTGTGGTGGACTG	20	60.16	55.00		
ATP Sulphurylase 1	ATPS1	269714	L:AATGGAAGTTCAAAAGGCACTC	22	59.64	40.91	100	1.98
			R:GTTGAAGAAGGGTGTTCCGTAG	22	60.03	50.00		
ATP Sulphurylase 2	ATPS2	1326	L:GATGATGTTGACGTGGTTGC	20	59.97	50.00	129	2.04
			R:GTAGGATGGACGAGGACAACTC	22	60.00	54.55		
APS Reductase 1*	APR1	35690	L:AGTTGGCACGTGAAAATGTG	20	59.62	45.00	127	1.99
			R:GGCTGTACGACAAGTAAGCAAG	22	59.11	50.00		
APS Reductase 2	APR2	24887	L:GAGAGTGCTGGAGTTCCTGTG	21	60.04	57.14	102	2.01
			R:GATGAAGTTGACAGTAGGGAAGC	23	59.30	47.83		
OAS Thiollyase 1*	OASTL1	22293	L:CAATACTTGAAACCTCGCAATC	22	58.77	40.91	156	1.92
			R:CGTCGATTAGAGAAGTGTCTGC	22	59.15	50.00		
OAS Thiollyase 2	OASTL2	31829	L:TGCTTCCTGACACTGGTGAG	20	60.02	55.00	109	1.92
			R:ATCCAACTGGGAGTGCTACG	20	60.13	55.00		
OAS Thiollyase 3	OASTL3	267987	L:GGATTGTTCGTCGGATCAAG	20	60.46	50.00	109	1.87
			R:GCCCTCCATCACATACAACAG	21	60.39	52.38		
Serine Acetyltrans-ferase 1	SAT1	38294	L:AGTTCTCGCGCATTACCTACAG	22	60.81	50.00	121	1.91
			R:CTCTCCAATAACGATCCCTGTG	22	60.87	50.00		
Serine Acetyltrans-ferase 1	SAT2	37497	L:CTCTCGGAGGTACGGGTAAAG	21	60.13	57.14	104	2.01
			R:GCCAACAAAGATATTTCCCAAG	22	59.84	40.91		
Serine Acetyltransf-erase 3	SAT3	16842	L:GAATGGCAATGTATGTGCAAAG	22	60.38	40.91	120	2.05
			R:TTTCACCAATCACCACTCCTC	21	59.96	47.62		
Sulphite Reductase	SiR	31984	L:CCGTGTACTCCTCAACAAGATG	22	59.66	50.00	101	1.96
			R:CCAATTCTGCCATGTAAGGAC	21	59.44	47.62		

*Indicates that the region of amplification includes an intron.
